# Identification and Expression Analysis of Wheat *TaGF14* Genes

**DOI:** 10.3389/fgene.2018.00012

**Published:** 2018-01-30

**Authors:** Jun Guo, Shuang Dai, Haosheng Li, Aifeng Liu, Cheng Liu, Dungong Cheng, Xinyou Cao, Xiusheng Chu, Shengnan Zhai, Jianjun Liu, Zhendong Zhao, Jianmin Song

**Affiliations:** ^1^National Engineering Laboratory for Wheat and Maize, Key Laboratory of Wheat Biology and Genetic Improvement in North Yellow and Huai River Valley, Ministry of Agriculture, Crop Research Institute, Shandong Academy of Agricultural Sciences, Jinan, China; ^2^Shandong Center of Crop Germplasm Resource, Shandong Academy of Agricultural Sciences, Jinan, China

**Keywords:** *Triticum aestivum*, 14-3-3, phylogenetic analysis, tissue-specific expression, starch biosynthesis

## Abstract

The 14-3-3 gene family members play key roles in various cellular processes. However, little is known about the numbers and roles of 14-3-3 genes in wheat. The aims of this study were to identify *TaGF14* numbers in wheat by searching its whole genome through blast, to study the phylogenetic relationships with other plant species and to discuss the functions of *TaGF14s*. The results showed that common wheat harbored 20 *TaGF14* genes, located on wheat chromosome groups 2, 3, 4, and 7. Out of them, eighteen *TaGF14s* are non-ε proteins, and two wheat *TaGF14* genes, *TaGF14i* and *TaGF14f*, are ε proteins. Phylogenetic analysis indicated that these genes were divided into six clusters: cluster 1 (*TaGF14d, TaGF14g, TaGF14j, TaGF14h, TaGF14c*, and *TaGF14n*); cluster 2 (*TaGF14k*); cluster 3 (*TaGF14b, TaGF14l, TaGF14m*, and *TaGF14s*); cluster 4 (*TaGF14a, TaGF14e*, and *TaGF14r*); cluster 5 (*TaGF14i* and *TaGF14f*); and cluster 6 (*TaGF14o, TaGF14p, TaGF14q*, and *TaGF14t*). Tissue-specific gene expressions suggested that all *TaGF14s* were likely constitutively expressed, except two genes, i.e., *TaGF14p* and *TaGF14f*. And the highest amount of *TaGF14* transcripts were observed in developing grains at 20 days post anthesis (DPA), especially for *TaGF14j* and *TaGF14l.* After drought stress, five genes, i.e., *TaGF14c*, *TaGF14d*, *TaGF14g*, *TaGF14h*, and *TaGF14j*, were up-regulated expression under drought stress for both 1 and 6 h, suggesting these genes played vital role in combating against drought stress. However, all the *TaGF14s* were down-regulated expression under heat stress for both 1 and 6 h, indicating *TaGF14s* may be negatively associated with heat stress by reducing the expression to combat heat stress or through other pathways. These results suggested that cluster 1, e.g., *TaGF14j*, may participate in the whole wheat developing stages, e.g., grain-filling (starch biosynthesis) and may also participate in combating against drought stress. Subsequently, a homolog of *TaGF14j*, *TaGF14-JM22*, were cloned by RACE and used to validate its function. Immunoblotting results showed that *TaGF14-JM22* protein, closely related to *TaGF14d*, *TaGF14g*, and *TaGF14j*, can interact with AGP-L, SSI, SSII, SBEIIa, and SBEIIb in developing grains, suggesting that *TaGF14s* located on group 4 may be involved in starch biosynthesis. Therefore, it is possible to develop starch-rich wheat cultivars by modifying *TaGF14s*.

## Introduction

The ubiquitous 14-3-3 proteins, as one of the families of regulatory proteins, have been found in all eukaryotic organisms and tissues. The family consists of dimeric α-helical pSer/Thr binding proteins that play key roles in various cellular processes, such as signal transduction, biotic and abiotic stress responses, and carbon and nitrogen metabolism, by mediating protein–protein interactions (Aitken et al., 1992; Fulgosi et al., 2002). However, little is known about the numbers and roles of 14-3-3 genes (*TaGF14s*) in wheat. Therefore, it is necessary to develop elite wheat cultivars to explore the numbers and to study the functions of *TaGF14s*.

Different species may have different numbers of *GF14s*. For example, humans have seven 14-3-3 genes (Iwata et al., 2000), while *Arabidopsis*, rice and maize have thirteen *GF14s* and two pseudogenes, eight *GF14s* and twelve *GF14s*, respectively (Wu et al., 1997; Rosenquist et al., 2001; Lai et al., 2004; Sehnke et al., 2006; Yao et al., 2007; Alexandrov et al., 2009), which suggested that plants maybe have more *GF14s* than animal. 14-3-3 proteins, binding a range of transcription factors and signaling proteins, have roles in regulating carbon and nitrogen metabolism, plant development, and biotic and abiotic stress responses (Roberts, 2000, 2003; Fulgosi et al., 2002; Maraschin et al., 2003). For example, *BdGF14f* were associated Cr and cold stresses in *Brachypodium distachyon* (Cao et al., 2016). Different 14-3-3 protein isoforms have different roles. For example, 14-3-3A processing and 14-3-3C isoform tissue specific expression are closely related to cell fate and initiation of specific cell type differentiation (Maraschin et al., 2003). And 14-3-3 proteins were also reported to be involved in starch biosynthesis in plants. For example, Alexander and Morris (2006) identified 54 14-3-3 binding proteins by MALDI-TOF MS, and the largest category was for carbohydrate metabolism, including plastidic enzymes for starch synthesis and modification. Out of them, four enzymes, i.e., GSBSI, SSI, SSII and SBEIIa were involved in starch biosynthesis. Presently, only four *GF14s* have been reported in common wheat (Ikeda et al., 2000; Yao et al., 2005; Wang et al., 2008). It is unknown whether there are more *GF14s* in common wheat than rice and maize. Due to the roles of *GF14* reported previously, it is necessary to study the 14-3-3 genes and their functions in wheat.

The allohexaploid bread wheat (*Triticum aestivum*, 2*n* = 6*x* = 42; genome AABBDD) is one of the largest crop worldwide. Due to two times of heterologous hybridization and two times of chromosome self-doubling, modern common wheat have a larger genome size (17 gigabase) than rice (466 megabases) and maize (2.3 gigabase) (Yu et al., 2002; Schnable et al., 2009; International Wheat Genome Sequencing Consortium [IWGSC], 2014). Because of its genome complexity and its big genome size, wheat chromosome sequencing is not possible in the last decade. However, with the advances of technologies, e.g., chromosome follow sorting and sequencing technology (next generation sequence/*de novo* assemble and pacbio), a reference genome of common wheat version TGACv1 is obtained by next generation sequence/*de novo* assembly (International Wheat Genome Sequencing Consortium [IWGSC], 2014), which is very attractive to wheat geneticists and breeders and highlights wheat genetic improvement.

In this study, we are aimed to identify *TaGF14* numbers in wheat by searching the wheat whole genome through blast, to study the phylogenetic relationships with other plant species and to discuss the functions of *TaGF14s*.

## Materials and Methods

### Plant Materials

The hard white winter wheat cultivar Jimai 22, released by our lab, was used in this study and was sown in a field at the Experimental Station of Shandong Academy of Agricultural Sciences (SAAS), Jinan, Shandong Province, China. The plot size was 12 m^2^. Soil fertility was high. Weeds and diseases were controlled. Developing wheat ears were tagged at the onset of anthesis. Endosperm tissue was obtained from developing wheat grains (at Z71 and Z75) taken from the mid-ear region of the head (Zadoks et al., 1974).

### RNA Extraction and Cloning of *TaGF14-JM22*

The total RNA was isolated from the developing grains or kernels at Zadok scale 71 according to the instructions of an RNeasy Plant Mini Kit (Qiagen, Germany). RNase-free DNase I (Promega, United States) was used to remove any contaminating genomic DNA. Quality and integrity of the total RNA were determined by running the appropriate amount of RNA in a formamide denaturing gel. *TaGF14-JM22* was cloned from wheat according to the methods described in the Supplementary Material. The cDNA sequence of *TaGF14-JM22* obtained was submitted to GenBank, and the accession number is GenBank JF957590. The 3D structure of *TaGF14-JM22* was predicted using the *ExPASy* proteomics online server and Swiss-Model^1^.

### Construction of the Phylogenetic Tree and Expression of the *TaGF14-JM22* Genes in Developing Grains

To determine the 14-3-3 gene numbers in wheat and to construct the phylogenetic tree of 14-3-3 genes from cereal crops and *Arabidopsis*, the coding sequence of *TaGF14-JM22*, cloned from wheat in this study, was used as the query to search the NCBI database^2^ and the genome sequence databases of Sorghum^3^, wheat^4^, and *Brachypodium*^5^ with a cut-off parameter of *E*-value ≤ 1E^-10^ for homologous *GF14s*. The phylogenetic tree was constructed using the maximum likelihood method with a Poisson distribution model and 1000 bootstrap replicates by MEGA 6.0 (Tamura et al., 2013) based on the amino acid sequences of 14-3-3 proteins with a cut-off value of 50% for the condensed tree. In addition, the silicon expression profiles of *TaGF14* in Root_Z13, Stem_Z30, Leaf_Z23, Spike_Z65, and the developing grains at 10, 20, and 30 days post anthesis (DPA) were obtained through *WheatExp* (Pearce et al., 2015) and analyzed. Data was analyzed with SAS software version 9.0. The mean expression values of every gene in different tissues were compared with each other, respectively. Duncan’s multiple range test was used to test for significant differences.

### Expression and Purification

For cloning in pET29c, the *TaGF14-JM22* sequence was amplified using the primers BamHI F and HindIII R. Amplicons were digested together with the pET29c vector and BamHI and HindIII enzymes at 37°C for 3 h. The digested products were purified and ligated together with T4 DNA ligase (Promega, United States) at 4°C overnight. The ligation mix was then transformed into *Escherichia coli* BL21 (DE3) for protein expression. The positive clones were screened for correct insertion by colony PCR and sequencing. The successful constructs were expected to express a *TaGF14-JM22* fusion protein with an S-tag at the N-terminus. The recombinant proteins were purified using the S-tag rEK Purification Kit (Novagen, United States) according to the manual’s protocol. For more details, please see the Supplementary Material.

### Amyloplast Isolation

The amyloplasts were isolated from the developing endosperm obtained from wheat grains (at Zadok scale 75) taken from the mid-ear region of the head as described by Tetlow et al. (2008). Starch granules were washed, and the granule-associated proteins, e.g., AGPase and GBSS, were extracted as described by Denyer et al. (1995). The protein content was measured using the Bio-Rad protein assay according to the manufacturer’s instructions and using thyroglobulin as a standard (Bio-Rad Lab., Canada).

### Preparation of Peptides and Antisera

Polyclonal antibodies of starch biosynthetic enzymes were raised in rabbits against synthetic peptides, which were derived from N-terminal sequences of wheat AGP-L (CIIDMNARIGRDVVISN, Ainsworth et al., 1995), AGP-S (AIIDKNARIGENVKIIN, Rösti and Denyer, 2007), SSI (APAQSPAPTQPPLPDAG, Li et al., 1999), SSII (ARVDDDAASARQPRARRG, Li et al., 1999), GBSSI (QDLSWKGPAKNWEDV, Vrinten and Nakamura, 2000), SBEI (VSAPRDYTMATAEDGV, Rahman et al., 2001), SBEIIa (AASPGKVLVPDGESDDLAS, Rahman et al., 2001), SBEIIb (AGGPSGEVMIPDGGSG, Regina et al., 2005), DE (SVGVGEDLPEGYEQM, Bresolin et al., 2006), and SP (NYDELMGSLEGNEGYGRADYFLV, Tickle et al., 2009). The antigen was prepared by coupling the synthesized peptide to keyhole limpet haemocyanin using the heterobifunctional reagent m-maleimidobenzoyl-N-hydroxysuccinimide ester (Tetlow et al., 2008).

### SDS-PAGE and Immunoblotting

The methods of SDS-PAGE and immunoblotting were according to Tetlow et al. (2008), for more detail, please see the Supplementary Materials. Gels were stained with a colloidal Coomassie Brilliant Blue G250 kit (Neuhoff et al., 1988).

## Results

### Numbers of *TaGF14* Genes and Phylogenetic Tree Construction

**FIGURE 1:**
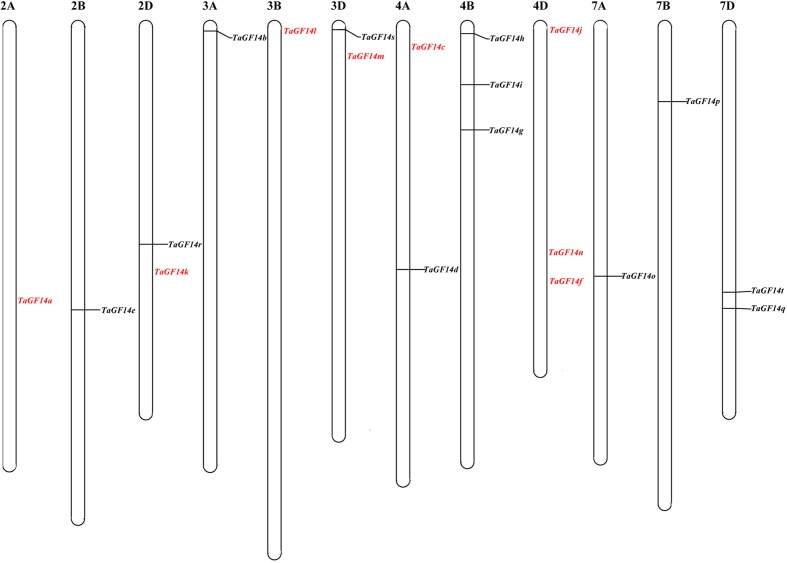
Chromosome localization of *TaGF14s* based on the reference sequence (TGACv1.0) of wheat genome (International Wheat Genome Sequencing Consortium [IWGSC], 2014). The text in *red* color presented that the genes were not physically mapped in the reference genome.

To explore the chromosomal locations and numbers of *TaGF14* in wheat, the complete coding sequence of *TaGF14-JM22* was used as the query to search the wheat whole-genome sequences published by IWGSC^6^. In total, 20 genes were obtained through Blast, and the coding sequences and chromosomal location of these genes are listed in Supplementary Table S1. In addition, these genes were located on wheat chromosome groups 2, 3, 4, and 7 (**Figure 1**). However, the 14-3-3 genes were not equally distributed on the wheat chromosome groups. In this study, eight genes were located on the wheat chromosome group 4 and the remaining 3 chromosome groups harbored equal numbers (four genes per group) of *TaGF14* genes.

**FIGURE 2:**
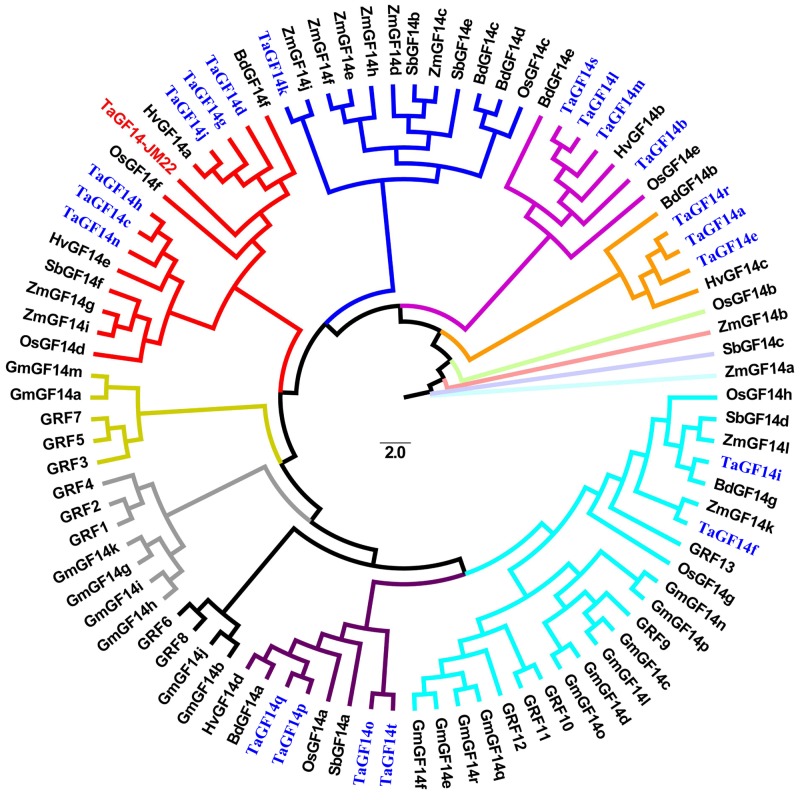
Phylogenetic analysis of *TaGF14s* in common wheat with 14-3-3 proteins in other plant species. A rooted phylogenetic tree based on the sequence alignment using the MEGA 6.0 software from the CLUSTALW multiple sequence alignment. The scale represents estimated branch length. The *TaGF14* genes from common wheat were marked in *blue* color.

To investigate the evolutionary relationship among *TaGF14-JM22* and other *GF14* genes and proteins derived from *Oryza sativa*, *B. distachyon*, *Zea mays*, *Hordeum vulgare*, and *Arabidopsis thaliana*, phylogenetic trees were constructed using the maximum likelihood method with a Poisson model and with 1000 bootstrap replicates by MEGA 6.0 (Tamura et al., 2013) based on the amino acid sequences of 14-3-3 proteins with a cut-off value of 50% for the condensed tree (**Figure 2**). The results showed that the 20 wheat *TaGF14s* could be divided into six clusters: cluster 1, including six genes (*TaGF14d, TaGF14g, TaGF14j, TaGF14h, TaGF14c*, and *TaGF14n*); cluster 2, including one gene (*TaGF14k*); cluster 3, including four genes (*TaGF14b, TaGF14l, TaGF14m*, and *TaGF14s*); cluster 4, including three genes (*TaGF14a, TaGF14e*, and *TaGF14r*); cluster 5, including two genes (*TaGF14i* and *TaGF14f*); and cluster 6, including four genes (*TaGF14o, TaGF14p, TaGF14q*, and *TaGF14t*). The results also showed that eighteen *TaGF14s* are non-ε proteins, except two wheat GF14 genes, *TaGF14i* and *TaGF14f*, which are ε proteins (**Figure 2**).

### Expression of *TaGF14s* in Wheat

**FIGURE 3:**
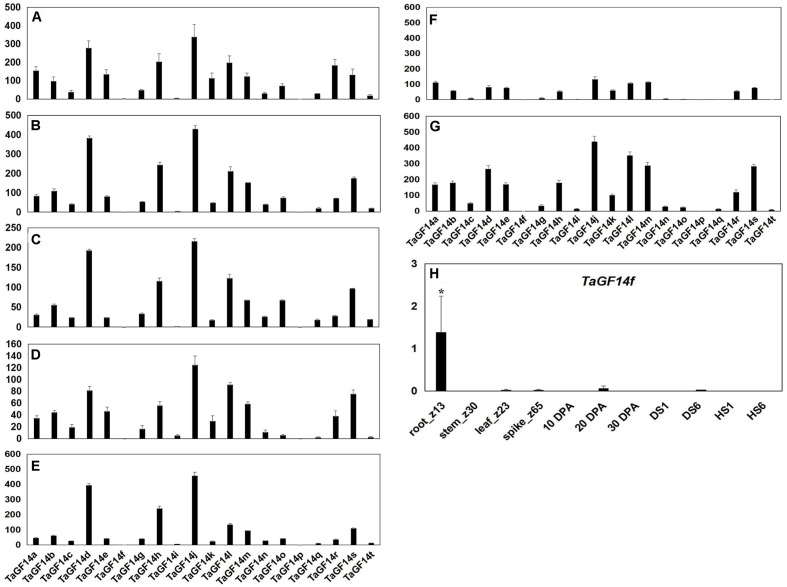
Detection of *TaGF14* transcripts by silicon expression profiles. **(A)** Root_Z13. **(B)** Stem_Z30. **(C)** Leaf_Z23. **(D)** Spike_Z65. **(E)** Developing grains_10 DPA. **(F)** Developing grains_20 DPA. **(G)** Developing grains_30 DPA. Bar represents the standard error. **(H)** The expression of *TaGF14f* was displayed in different tissues. ^∗^At the top of each column indicates significant difference at *P* = 0.05.

In order to investigate the gene expression levels of *TaGF14* in the wheat root, stem, leaf, spike and developing grains at 10, 20, and 30 DPA, the silicon expression dataset was downloaded from *WheatExp*. As shown in **Figure 3**, it appeared that all *TaGF14s* were constitutively expressed, except two genes, i.e., *TaGF14p*, which was not observed, and *TaGF14f*, which showed tissue-specific expression in the root (**Figure 3H** and Supplementary Figure S6). In addition, *TaGF14i* was also likely expressed in a tissue-specific manner in stem_z30, the developing grains at 20 and 30 DPA. *TaGF14o*, *TaGF14q*, and *TaGF14t*, which belong to cluster 6, were expressed less in developing grains than in other investigated tissues (Supplementary Figure S6). These results indicated that *TaGF14j*, *TaGF14l*, and *TaGF14i* may play important role in the wheat grain-filling stage.

**FIGURE 4:**
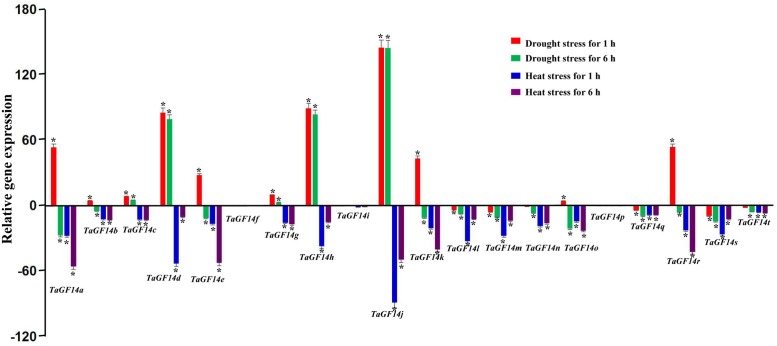
Wheat *TaGF14s* expression induced by drought and heat. Red, green, blue, and purple column represent drought stress for 1 h, drought stress for 6 h, heat stress for 1 h, and heat stress for 6 h. Bar represents the standard error. ^∗^At the top of each column indicates significant difference at *P* = 0.05.

**FIGURE 5:**
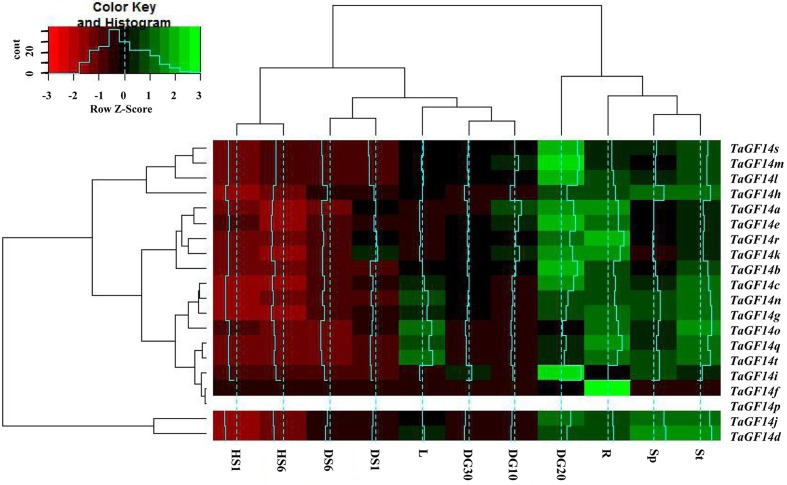
Heatmap of *TaGF14s* drawn with software R program (gplot) based on its expression data in different tissues or samples treated with drought and heat stresses. St, Stem_Z30. Sp, Spike_Z65. R, Root_Z13. L, Leaf_Z23. DG10, Developing grains_10 DPA. DG20, Developing grains_20 DPA. DG30, Developing grains_30 DPA.

In addition, the gene expressions of *TaGF14s* were also determined in the wheat seedling stage treated with drought stress and heat stress. The results showed that five genes, i.e., *TaGF14c*, *TaGF14d*, *TaGF14g*, *TaGF14h*, and *TaGF14j*, were up-regulated expression under drought stress for both 1 and 6 h (**Figure 4**), suggesting these genes played vital role in combating against drought stress. However, all the *TaGF14s* were down-regulated expression under heat stress for both 1 and 6 h, which indicated that *TaGF14s* may be negatively associated with heat stress by reducing the expression to combat heat stress or through other pathways. Furthermore, the heatmap of *TaGF14s* were also drawn based on the gene expression data of *TaGF14s*. The results showed that *TaGF14s* in Root_Z13, Stem_Z30, Spike_Z65 and the developing grains at 20 DPA had the similar gene expression, while the rest had the similar gene expression pattern (**Figure 5**). And the *TaGF14s* clustered into three clusters based on gene expression in different samples or tissues, i.e., CL1, CL2, and CL3. And *TaGF14h*, *TaGF14l*, *TaGF14m*, and *TaGF14s* belonged to CL1. *TaGF14d* and *TaGF14j* belonged to CL3, while the rest belonged to CL2. These results suggested that *TaGF14d* and *TaGF14j*, both constitutively expressed, may participate in the whole wheat developing stages, e.g., grain-filling (starch biosynthesis) and may also participate in combating against drought stress.

### Cloning and Sequence Analysis of *TaGF14-JM22*

To validate the gene function of *TaGF14j*, a homologous gene, *TaGF14-JM22* was cloned and used for further analysis. The full-length cDNA of *TaGF14-JM22*, containing 786 nucleotides, was obtained using the RACE-PCR technique (Supplementary Table S3 and Supplementary Figures S1, S2) and submitted to GenBank (Accession number: JF957590). Multiple alignments showed that this sequence shared high identity with 14-3-3 proteins from other species ranging from 31 to 98% (Supplementary Figure S3), e.g., 98% identity with *Brachypodium* (*BdGF14f*) and *Oryza* (*OsGF14f*) and 31% identity with *Oryza* (*OsGF14h*). *TaGF14-JM22* was predicted to encode 261 amino acids (AA), with a predicted molecular mass of 29.27 kDa and an isoelectric point (pI) of 4.82. Structure analysis revealed that the predicted *TaGF14-JM22* protein contained two 14-3-3 protein signatures and six functional motifs (Supplementary Table S2), such as a cAMP- (or cGMP-) dependent protein kinase phosphorylation site and a tyrosine kinase phosphorylation site, which were highly conserved in 14-3-3 homologs. Based on a WoLF PSORT analysis^7^, *TaGF14-JM22* was located in the plasma membrane or nuclear plasma membrane. In addition, the three-dimensional (3D) structure prediction was analyzed by comparative protein modeling. The coding sequence of *TaGF14-JM22* was submitted to the Swiss-Model online server^8^, and six 14-3-3-like proteins with sequence similarities of 90.60, 90.34, 90.17, 85.83, 84.86, and 84.52% were selected as templates to build models. Subsequently, nine models were generated using the abovementioned 14-3-3 proteins as models for the Swiss-Model homology modeling (Supplementary Figure S5). In addition, the QMEAN Z-score evaluations for the models were -1.12, -0.95, -1.50, -1.03, -0.50, -0.97, -1.33, -1.39, and -1.75, respectively, showing that the predicted models were of good quality. Furthermore, the phylogenetic results indicated that *TaGF14-JM22*, cloned in this study and belonging to non-ε protein, was closely related to three wheat genes (*TaGF14d*, *TaGF14g*, and *TaGF14j*) as well as *OsGF14f* and *HvGF14f* (**Figure 2** and Supplementary Figure S3).

### Validation the Function of *TaGF14-JM22* in Developing Grains

To validate *TaGF14-JM22* similar to *TaGF14j* participating in starch biosynthesis in developing grains, the coding sequence of *TaGF14-JM22* was sub-cloned into pET29c. After induction by 1 mM IPTG at 37°C for 1, 3, 5, and 7 h, the highest expression occurred with 1 mM IPTG in both 5 and 7 h inductions at 37°C. SDS-PAGE was used for induction and purification of the *TaGF14-JM22* protein. The protein with the highest abundance was found in the *E. coli* extracts. The molecular mass of the induced protein was about 29 kDa, which was in accordance with the predicted amino acid sequence (Supplementary Figure S4).

**FIGURE 6:**
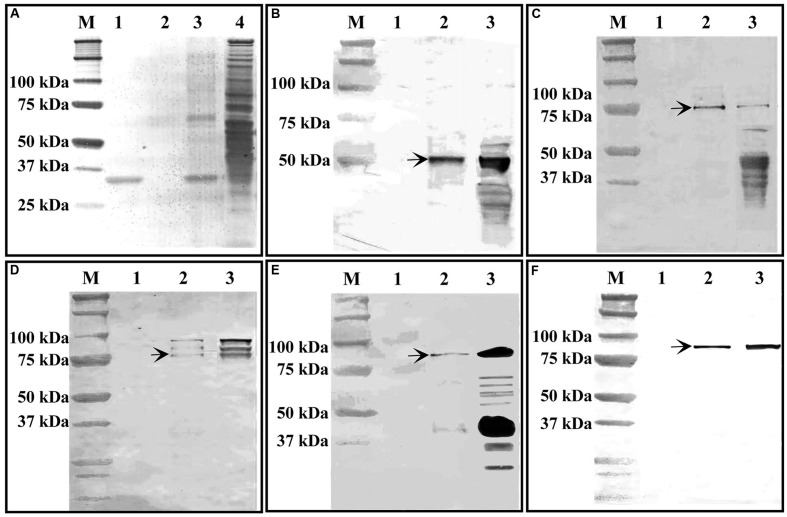
Interaction between GF14-JM22 protein and starch biosynthetic enzymes revealed by immunoblotting assays. M, Protein markers. **(A)** Gel image of SDS-PAGE. 1, Recombinant GF14-JM22 protein binding to S-protein agarose resin. 2, Blank control. 3, Recombinant GF14-JM22 protein through affinity chromatography. 4, Wheat amyloplast extracts. **(B–F)**, Gel images of SH2-antibody, SSI-antibody, SSII-antibody, SBEIIa-antibody and SBEIIb-antibody, respectively. 1, Blank control. 2, Recombinant GF14-JM22 protein immunoblotting through affinity chromatography. 3, Wheat amyloplast extracts immunoblotting.

The purified recombinant *TaGF14-JM22* protein was bound to S-protein agarose as a biochemical bait and then incubated with wheat amyloplast extract. Protein–protein interactions between the *TaGF14-JM22* protein and ten key starch biosynthetic enzymes from amyloplasts, i.e., AGP-L, AGP-S, SSI, SSII, GBSSI, SBEI, SBEIIa, DE, SBEIIb, and SP, were investigated and analyzed by SDS-PAGE and western blotting. As shown in **Figure 6**, BSA, as a control, could not bind any starch biosynthetic enzymes, but protein–protein interactions between the *TaGF14-JM22* protein and starch biosynthetic enzymes were observed. AGP-L, SSI, SSII, SBEIIa, and SBEIIb interacted with the *TaGF14-JM22* protein (**Figure 6**). However, AGP-S, GBSSI, SBEI, DE, and SP could not interact with the *TaGF14-JM22* protein. These results suggested that *TaGF14-JM22* indeed participated in starch biosynthesis by binding to biosynthetic enzymes.

## Discussion

The family of 14-3-3 proteins is one of the families of regulatory proteins in plants (Aitken et al., 1992). Previous studies showed that plants have more 14-3-3 genes than animals. For example, human has seven 14-3-3 genes, while *Arabidopsis* and maize have thirteen 14-3-3 genes and twelve 14-3-3 genes, respectively (Wu et al., 1997; Iwata et al., 2000; Rosenquist et al., 2001). However, the number of 14-3-3 genes in common wheat and their relationships with other species are still unknown. In the present study, it was determined by Blastn against the whole genome sequences of Chinese Spring wheat released by International Wheat Genome Sequencing Consortium [IWGSC], 2014. The results indicated that common wheat harbored 20 *GF14s* (Supplementary Table S1 and **Figure 2**), which was much more than rice and *Brachypodium* (Wu et al., 1997; Iwata et al., 2000; Rosenquist et al., 2001). Of all the genes, eight (40%) were located on wheat chromosome group 4 (Supplementary Table S1). In addition, the phylogenetic tree was constructed based on 14-3-3 protein sequences, which revealed that most of the TaG*F14*s, including five clusters (clusters 1–5), are non-ε proteins, except cluster 6 (*TaGF14f* and *TaGF14i*) which are ε proteins (**Figure 2** and Supplementary Table S1).

The 14-3-3 proteins play important roles in diverse cellular processes by mediating protein-protein interactions in plants (Fulgosi et al., 2002). Previous studies indicated that *HvGF14a* was a protein induced by powdery mildew fungus, suggesting that it was involved in plant resistance to fungus infection in *H. vulgare* (Brandt et al., 1992). In *Brachypodium*, *BdGF14f* was significantly induced by Cr and cold stress (Cao et al., 2016). In addition, *OsGF14f* was constitutively expressed in rice (Yao et al., 2007). Previous studies indicated that starch was synthesized through the coordinated interactions of a suite of biosynthetic enzymes in plants (Zeeman et al., 2010). However, whether 14-3-3 as a regulatory protein involved in starch biosynthesis was known in wheat. And very little was also known about the functions of 14-3-3 proteins in wheat. In this study, *TaGF14-JM22*, which is similar to *TaGF14d*, *TaGF14g* and *TaGF14j*, the most highly expressed genes among the twenty *TaGF14s* in developing wheat grains, was used to investigate the protein-protein interactions between 14-3-3s and ten key starch biosynthetic enzymes, i.e., AGP-L, AGP-S, SSI, SSII, GBSSI, SBEI, SBEIIa, DE, SBEIIb, and SP, during grain filling by a immunoblotting assay. The results showed that five enzymes, i.e., AGP-L, SSI, SSII, SBEIIa, and SBEIIb, interacted with the *TaGF14-JM22* protein, while the rest of the enzymes did not (**Figure 6**), suggesting that *TaGF14d*, *TaGF14g*, and *TaGF14j* may be involved in starch biosynthesis through protein–protein interactions. In barley, 14-3-3 proteins were reported to interact with four starch biosynthetic enzymes, i.e., GSBSI, SSI, SSII and SBEIIa in developing grains (Alexander and Morris, 2006), which were also clearly detected in our study. In addition, two starch biosynthetic enzymes, i.e., DE and SBEIIb, also interacted with 14-3-3 proteins in developing wheat grains, which was firstly reported in this study and may be unique to wheat, considering the fact that wheat harbors more 14-3-3s than other species (**Figures 2**, **6**). In addition, the results showed that *TaGF14s* in Leaf_Z23, the developing grains at 10 and 30 DPA, drought stress and heat stress had the similar gene expressions, which can be explained by the fact that wheat production was usually affected by heat and drought stress, especially in the grain-filling stages (Skylas et al., 2002; Farooq et al., 2011). And in **Figures 2**, **3**, the results showed that *TaGF14-JM22*, *TaGF14d*, *TaGF14g*, and *TaGF14j* were closely related to *HvGF14a* (a pathogen-related protein), *BdGF14f*, induced by Cr and cold stress, and *OsGF14f*, and were constitutively expressed in wheat (Yao et al., 2007; Cao et al., 2016). Therefore, we speculated that *TaGF14d*, *TaGF14g*, and *TaGF14j* may also have the similar functions, e.g., resistance to pathogen and Cr stress, with other plant species. Furthermore, *Arabidopsis GRF6* was linked to the “stay green” phenotype and drought tolerance by cotton transformation experiments (Yan et al., 2004). Our results indicated that the *TaGF14*s, closely related to *GRF6*, on group 7 that belong to cluster 6 may also be linked to the “stay green” phenotype (**Figure 2**). So, in our next project, the functions of *TaGF14s*, especially *TaGF14s* located on group 4 and group 7 will be further analyzed by gene overexpressing or gene silencing in wheat.

## Author Contributions

JS and JG conceived and designed the experiments. JS, JG, SD, and HL performed the experiments. JG, CL, XsC, and JS analyzed the data. DC, AL, XyC, SZ, ZZ, and JL contributed reagents/materials/analysis tools. JG and JS wrote the paper.

## Conflict of Interest Statement

The authors declare that the research was conducted in the absence of any commercial or financial relationships that could be construed as a potential conflict of interest.

## Footnotes

1 https://swissmodel.expasy.org/

2 https://www.ncbi.nlm.nih.gov/

3 http://www.plantgdb.org/SbGDB/

4 https://urgi.versailles.inra.fr/blast/

5 http://www.brachypodium.org/

6 https://urgi.versailles.inra.fr/blast/

7 https://www.genscript.com/wolf-psort.html

8 https://swissmodel.expasy.org/
