# Supplementary material for: Identification and Expression Analysis of Wheat TaGF14 Genes
Source: Front Genet. 2018 Jan 30;9:12. doi: 10.3389/fgene.2018.00012 (PMC5797578; doi:10.3389/fgene.2018.00012)
Supplement: Supplementary file 1 [file Table_1.pdf]

Supplementary Table S1. The CDS of 14-3-3 genes and their deduced proteins in 8 representative species

Lineage	Organism	Nomenclature	Locus name	Chr.	Subgroup	CDS Length	Protein Length
Monocots	*Brachypodium distachyon*	***BdGF14a***	***Bradi3g38640***	Chr 03	non-ε	789	262
		BdGF14b	***Bradi5g12510***	Chr 05	non-ε	789	262
		BdGF14c	***Bradi3g36480***	Chr 03	non-ε	771	256
		BdGF14d	***Bradi4g16640***	Chr 04	non-ε	789	266
		BdGF14e	***Bradi3g46960***	Chr 03	non-ε	801	262
		BdGF14f	***Bradi1g11290***	Chr 01	non-ε	792	263
		BdGF14g	***Bradi4g13970***	Chr 04	ε	897	298
	Oryza sativa	***OsGF14a***	***Os08g37490***	Chr 08	non-ε	795	246
		OsGF14b	***Os04g38870***	Chr 04	non-ε	789	262
		OsGF14c	***Os08g33370***	Chr 08	non-ε	771	256
		OsGF14d	***Os11g34450***	Chr 11	non-ε	798	265
		OsGF14e	***Os02g36974***	Chr 02	non-ε	789	262
		OsGF14f	***Os03g50290***	Chr 03	non-ε	783	260
		OsGF14g	***Os01g11110***	Chr 01	ε	612	203
		OsGF14h	***Os11g39540***	Chr 11	ε	693	230
	Triticum aestivum	***TaGF14a***	***Traes_2AL_197BC062C***	Chr 2AL	non-ε	792	263
		TaGF14b	***Traes_3AS_8A727B48F***	Chr 3AS	non-ε	789	262
		TaGF14c	***Traes_4AS_2FF2E3CAF***	Chr 4AS	non-ε	801	266
		TaGF14d	***Traes_4AL_6B5C89E7C***	Chr 4AL	non-ε	786	261
		TaGF14e	***Traes_2BL_1B2A85367***	Chr 2BL	non-ε	792	263
		TaGF14f	***Traes_4DL_9C6C9CB1A***	Chr 4DL	-	-	-
		TaGF14g	***Traes_4BS_342A62CE6***	Chr 4BS	non-ε	801	266
		TaGF14h	***Traes_4BS_F7F9DE1D4***	Chr 4BS	non-ε	786	261
		TaGF14i	***Traes_4BS_D9C942644***	Chr 4BS	ε	750	249
		TaGF14j	***Traes_4DS_8D47B4C37***	Chr 4DS	non-ε	786	261

		TaGF14k	***Traes_2DL_1D912517E***	**Chr 2DL**	**non-ε**	**552**	**183**
		TaGF14l	***Traes_3B_3BF024700350CFD_c1***	**Chr 3BS**	**non-ε**	**789**	**262**
		TaGF14m	***Traes_3DS_63471506E***	**Chr 3DS**	**non-ε**	**792**	**263**
		TaGF14n	***Traes_4DL_BCCE5731E***	**Chr 4DL**	**non-ε**	**801**	**266**
		TaGF14o	***Traes_7AL_AA81EE544***	**Chr 7AL**	**non-ε**	**693**	**230**
		TaGF14p	***Traes_7BS_C7A3E1534***	**Chr 7BS**	**non-ε**	**576**	**191**
		TaGF14q	***Traes_7DL_73928596C***	**Chr 7DL**	**non-ε**	**549**	**182**
		TaGF14r	***Traes_2DL_21639EB55***	**Chr 2DL**	**non-ε**	**327**	**108**
		TaGF14s	***Traes_3DS_CDC0F6925***	**Chr 3DS**	**non-ε**	**324**	**107**
		TaGF14t	***Traes_7DL_18287B4F2***	**Chr 7DL**	**non-ε**	**285**	**94**
	Zea mays	***ZmGF14a***	***GRMZM2G102499***	**Chr 02**	**non-ε**	**837**	**278**
		ZmGF14b	***GRMZM2G078641***	**Chr 10**	**non-ε**	**786**	**261**
		ZmGF14c	***GRMZM2G091155***	**Chr 04**	**non-ε**	**771**	**256**
		ZmGF14d	***AC217050.4_FG006***	**Chr 01**	**non-ε**	**771**	**256**
		ZmGF14e	***GRMZM2G153823***	**Chr 01**	**non-ε**	**774**	**257**
		ZmGF14f	***GRMZM2G107756***	**Chr 06**	**non-ε**	**1029**	**342**
		ZmGF14g	***GRMZM2G106424***	**Chr 04**	**non-ε**	**807**	**268**
		ZmGF14h	***GRMZM2G006207***	**Chr 06**	**non-ε**	**765**	**254**
		ZmGF14i	***GRMZM2G408768***	**Chr 02**	**non-ε**	**801**	**266**
		ZmGF14j	***GRMZM5G8660***	**Chr 04**	**non-ε**	**759**	**252**

			82				
		ZmGF14k	*GRMZM2G140545*	Chr 04	non-ε	780	259
		ZmGF14l	*GRMZM2G145213*	Chr 04	ε	747	248
	Hordeum vulgare	*HvGF14a*	*Hv14-3-3a*	Chr 04	non-ε	786	261
		HvGF14b	*Hv14-3-3b*	Chr 03	non-ε	789	262
		HvGF14c	*Hv14-3-3c*	Chr 02	non-ε	792	263
		HvGF14d	*Hv14-3-3d*	Chr 07	non-ε	780	259
		HvGF14e	*Hv14-3-3e*	Chr 04	non-ε	801	266
	Sorghum bicolor	*SbGF14a*	*Sb07g029110*	Chr 07	non-ε	780	259
		SbGF14b	*Sb07g020990*	Chr 07	non-ε	771	256
		SbGF14c	*Sb06g019100*	Chr 06	non-ε	786	261
		SbGF14d	*Sb05g024160*	Chr 05	ε	744	247
		SbGF14e	*Sb07g025680*	Chr 07	non-ε	762	253
		SbGF14f	*Sb05g021020*	Chr 05	non-ε	798	265
Dicots	*Arabidopsis thaliana*	*GRF1*	*AT4G09000*	Chr 04	non-ε	957	318
		GRF2	*AT1G78300*	Chr 01	non-ε	780	259
		GRF3	*AT5G38480*	Chr 05	non-ε	768	255
		GRF4	*AT1G35160*	Chr 01	non-ε	888	295
		GRF5	*AT5G16050*	Chr 05	non-ε	807	268
		GRF6	*AT5G10450*	Chr 05	non-ε	798	273
		GRF7	*AT3G02520*	Chr 03	non-ε	822	265
		GRF8	*AT5G65430*	Chr 05	non-ε	783	260
		GRF9	*AT2G42590*	Chr 02	ε	831	276
		GRF10	*AT1G22300*	Chr 01	ε	765	254
		GRF11	*AT1G34760*	Chr 01	ε	768	255
		GRF12	*AT1G26480*	Chr 01	ε	807	268
		GRF13	*AT1G78220*	Chr 01	ε	738	245
	Glycine max	*GmGF14a*	*Glyma08g47900*	Chr 08	non-ε	783	260
		GmGF14b	*Glyma04g09820*	Chr 04	non-ε	753	250
		GmGF14c	*Glyma05g29080*	Chr 05	ε	780	259
		GmGF14d	*Glyma13g36690*	Chr 13	ε	786	261
		GmGF14e	*Glyma01g07070*	Chr 01	ε	780	259
		GmGF14f	*Glyma02g12930*	Chr 02	ε	780	259
		GmGF14g	*Glyma02g37120*	Chr 02	non-ε	789	262
		GmGF14h	*Glyma06g10650*	Chr 06	non-ε	840	279

		GmGF14i	***Glyma04g10820***	**Chr 04**	**non-ε**	**867**	**288**
		GmGF14j	***Glyma06g09890***	**Chr 06**	**non-ε**	**753**	**250**
		GmGF14k	***Glyma14g35410***	**Chr 14**	**non-ε**	**945**	**314**
		GmGF14l	***Glyma08g12220***	**Chr 08**	**ε**	**780**	**259**
		GmGF14m	***Glyma18g53610***	**Chr 18**	**non-ε**	**774**	**257**
		GmGF14n	***Glyma12g35820***	**Chr 12**	**ε**	**798**	**265**
		GmGF14o	***Glyma12g33800***	**Chr 12**	**ε**	**786**	**261**
		GmGF14p	***Glyma13g34580***	**Chr 13**	**ε**	**792**	**263**
		GmGF14q	***Glyma07g35240***	**Chr 07**	**ε**	**780**	**259**
		GmGF14r	***Glyma20g03120***	**Chr 20**	**ε**	**783**	**260**
