# Supplementary material for: Identification and Expression Analysis of Wheat TaGF14 Genes
Source: Front Genet. 2018 Jan 30;9:12. doi: 10.3389/fgene.2018.00012 (PMC5797578; doi:10.3389/fgene.2018.00012)
Supplement: Supplementary file 2 [file Table_2.pdf]

Supplementary Table S2. Predicted prosites of the predicted structure of the 14-3-3 protein from developing wheat endosperms according to the ProtParam tool.

Prosite	Pattern	Location and sequence
N-glycosylation site	N[^P][ST][^P]	^{182}NFSV;^{233}NLTL
cAMP- and cGMP- dependent protein kinase phosphorylation site	[RK]{2}.[ST]	^{62}RRAS
Protein kinase C phosphorylation site	[ST].[RK]	^{65}SWR; ^{87}SIK; ^{219}SYK
Casein kinase II phosphorylation site	[ST].{2}[DE]	^{2}STAE; ^{7}TREE; ^{44}TVEE; ^{70}SSIE; ^{87S}IKE; ^{93}SRIE; ^{119}TAAE; ^{143}SGAE; ^{160}SAQD; ^{214}TLGE; ^{219}SYKD; ^{254}SKPE
Tyrosine kinase phosphorylation site	[RK].{2,3}[DE].{2,3}Y	^{129}KMKGDYHRY
N-myristoylation site	G[^EDRKHPFYW].{2}[STAGCN][^P]	^{60}GARRAS; ^{178}GLALNF
14-3-3 proteins signature 1	[RA]NL[LIV]S[VG][GA]Y[KN]N[IVA]	^{48}RNLLSVAYKNV
14-3-3 proteins signature 2	YK[DE][SG]TLI[IML]QL[LF][RHC]DN[LF]T[LS]W[TANS][SAD]	^{220}YKDSTLIMQLLRDNLTLWTS
