# Supplementary material for: Identification and Expression Analysis of Wheat TaGF14 Genes
Source: Front Genet. 2018 Jan 30;9:12. doi: 10.3389/fgene.2018.00012 (PMC5797578; doi:10.3389/fgene.2018.00012)
Supplement: Supplementary file 3 [file Table_3.pdf]

Supplementary Table S3. The primers used in this study.

Primer	Primer sequence (5'-3')
UPM_long	CTAATACGACTCACTATAGGGCAAGCAGTGGTATCAACGCAGAGT
UPM_short	CTAATACGACTCACTATAGGGC
NUP	AAGCAGTGGTAACAACGCAGAGT
GSP1	TAAGGAGCGGCTCCCTCAGGTCGAG
GSP2	ACCTT/CGTCCCCTCTGCCACTGCTGCA
BamHI F	CCAGAGGAAAGGATCCGAACTAGTT
HindIII R	GCCAAGCTTTGCCCCTCTCCCTCAG
