# Supplementary material for: Identification and Expression Analysis of Wheat TaGF14 Genes
Source: Front Genet. 2018 Jan 30;9:12. doi: 10.3389/fgene.2018.00012 (PMC5797578; doi:10.3389/fgene.2018.00012)
Supplement: Supplementary file 4 [file Presentation_1.pdf]

Identification and expression analysis of wheat TaGF14 genes

Jun Guo[1, **], Shuang Dai[2, **], Haosheng Li[1, **], Cheng Liu[1], Dungong Cheng[1], Aifeng Liu[1], Xinyou Cao[1], Xiusheng Chu[1], Shengnan Zhai[1], Jianjun Liu[1], Zhendong Zhao[1], Jianmin Song[1, *]

[1] National Engineering Laboratory for Wheat and Maize & Key Laboratory of Wheat Biology and Genetic Improvement in North Yellow and Huai River Valley, Ministry of Agriculture, Crop Research Institute, Shandong Academy of Agricultural Sciences (SAAS), No. 202, Gongye North Road, Jinan 250100, China

[2] Shandong Center of Crop Germplasm Resource, Shandong Academy of Agricultural Sciences (SAAS), Jinan 250100, China

[*]Correspondence:

J Song

Tel.: +86-531-8317 9561

E-mail: wheat_breeding2016@163.com

** Authors contributed equally to this work

Materials and methods

RNA extraction and cloning of *TaGF14-JM22*

The total RNA was isolated from the developing grains or kernels at Zadok scale 71 according to the instructions of an RNeasy Plant Mini Kit (Qiagen, Germany). RNase-free DNase I (Promega, USA) was used to remove any contaminating genomic DNA. Quality and integrity of the total RNA were determined by running the appropriate amount of RNA in a formamide denaturing gel. First-strand cDNA was synthesized according to the instructions of EasyScript First-Strand cDNA Synthesis SuperMix (TransGen, China). The first-strand cDNA was then directly used as the template for the cloning of *TaGF14-JM22*.

To obtain the complete coding sequence (CDS) of wheat *TaGF14-JM22*, 3'-RACE and 5'-RACE were performed according to the instructions of a SMART™ RACE cDNA Amplification Kit (Clontech, USA). The nucleotide sequence of the barley *Hvl4-3-3a* gene (GenBank: X62388) was initially used as the query sequence to search the wheat EST database in GenBank using Blastn. Two homologous wheat EST sequences (KC121320 and AY386126) were retrieved from the GenBank database. In addition, the gene-specific primers GSP1 and GSP2 (Supplementary Table S3) were designed based on the conserved sequences aligned by X62388, KC121320 and AY386126 (Supplementary Figure S1). Then, the 5'-sequence and 3'-sequence of *TaGF14-JM22* were obtained by primer pairs GSP1 and NUP, GSP1 and UPM (Supplementary Table S3), respectively. A nested PCR was performed in the 5′-RACE and 3′-RACE methods. The PCR amplicons were separated by 1.5% agarose gel electrophoresis. In addition, the target bands were excised from the gels, purified, ligated into the pMD19-T vector (TransGen, China), and sequenced by Sangon Company (Shanghai, China). Subsequently, the complete open reading frame (ORF) of *TaGF14-JM22* was obtained by assembling the 5'-sequence and 3'-sequence of *TaGF14-JM22* using DNAMAN software and Blast software online.

Expression and purification

The positive colony was inoculated into 100 ml LB/ampicillin medium and grown to

an $O.D._{600}$ of 0.6 in a shaking incubator set at 37 °C and 250 rpm. After 1, 3, 5, and 7 h inductions with IPTG, the cell culture was harvested by centrifugation (Eppendorf 5427R, Germany) at 4000×g for 20 minutes at 4 °C. The pellet was resuspended in 10 ml of 1×PBS buffer containing 137 mM NaCl, 2.7 mM KCl, 10 mM Na_2HPO_4, 1.8 mM KH_2PO_4, and buffer (pH 7.4). The resuspended pellet was centrifuged at 4000×g for 15 minutes at 4 °C. The pellet was washed again with 1×PBS. The recombinant proteins were extracted after lysis using BugBuster Protein Extraction Reagent (Novagen, USA) according to the manual's protocol.

SDS-PAGE and immunoblotting

S-protein agarose slurry (500 µl) with 250 µg of purified S-tagged recombinant proteins and 500 µl of slurry without bound proteins as a control were individually mixed with 500 µl of 1.5% BSA (Sigma) to block antibody binding by incubation for 60 min at 4 °C. Two millilitres of wheat amyloplast preparation (0.3 mg ml^{-1}) and 30 µl of protease inhibitor cocktail (Sigma) were added to each reaction and shaken at room temperature for 2 h. Both were centrifuged at 500×g for 5 min at 4 °C, and the supernatants were carefully removed. The beads were moved to a disposable plastic column and washed with 150 ml of washing buffer (20 mM L^{-1} Tris-HCl pH 7.5, 150 mM L^{-1} NaCl, 0.1% Triton X-100). Then, the beads were boiled in 500 µl washing buffer and 250 µl 5×SDS-PAGE loading buffer at 95 °C for 7 min (the beads were inverted every 2 min). Afterward, 160 µl of the supernatant was removed and boiled with 40 µl 5×SDS-PAGE loading buffer at 95 °C for 5 min. Protein samples were separated on 10% SDS-PAGE. Gels were stained with a colloidal Coomassie Brilliant Blue G250 kit (Neuhoff et al. 1988).

For immunoblot analysis, samples were processed as described by Tetlow et al. (2008). Briefly, the samples were transblotted onto nitrocellulose membranes, blocked with 1.5% BSA, and exposed to antibodies. The various antisera were utilized in immunoblot analyses with the following dilutions: S-tag antibody 1:5,000; anti-AGP-L, anti-AGP-S, anti-SSI, anti-SSII and anti-GBSSI, 1:3,000; anti-SBEI, anti-SBEIIa, and anti-DE, 1:5,000; anti-SBEIIb and anti-SP, 1:2,000. In addition, the

bound antibodies were determined using alkaline phosphatase-conjugated goat anti-rabbit IgG on a 5-bromo-4-chloro-3-indolyl phosphate/nitro blue tetrazolium liquid substrate system (Sigma).

Supplementary Figures

Supplementary Figure S1. Multiple sequence alignment of amino acid sequences of X62388, KC121320 and AY386126 and primer design

Supplementary Figure S2. PCR amplification of *TaGF14-JM22*. 1, The full length of *TaGF14-JM22*. 2, 3' RACE amplification of *TaGF14-JM22*. 3, 5' RACE amplification of *TaGF14-JM22*.

Supplementary Figure S3. Multiple sequence alignment of the amino acid sequences of *TaGF14-JM22* (GenBank JF957590) with four other species, i.e., barley (*HvGF14a*, X62388), rice (*OsGF14f*, AK103065), maize (*ZmGF14g*, *GRMZM2G106424*) and *Brachypodium* (*BdGF14f*, *Bradi1g11290*). Protein sequences were aligned using the CLUSTALW alignment algorithm. Sequences were shaded using the BoxShade program. Identical and conserved residues are shaded in black and grey, respectively. In the picture, *TaGF14-JM22* shows high identity with other *GF14s*.

Supplementary Figure S4. Expression and purification of *TaGF14-JM22* in *E coli*. M, Protein markers. 1, Bacterial proteins form BL21 transformed with pMD19-T-*TaGF14-JM22* uninduced with IPTG, lanes 2 to 5, Bacterial proteins from BL21 transformed with pMD19-T-*TaGF14-JM22* induced with 1 mM IPTG at 7 °C for 1, 3, 5 and 7 h, respectively.

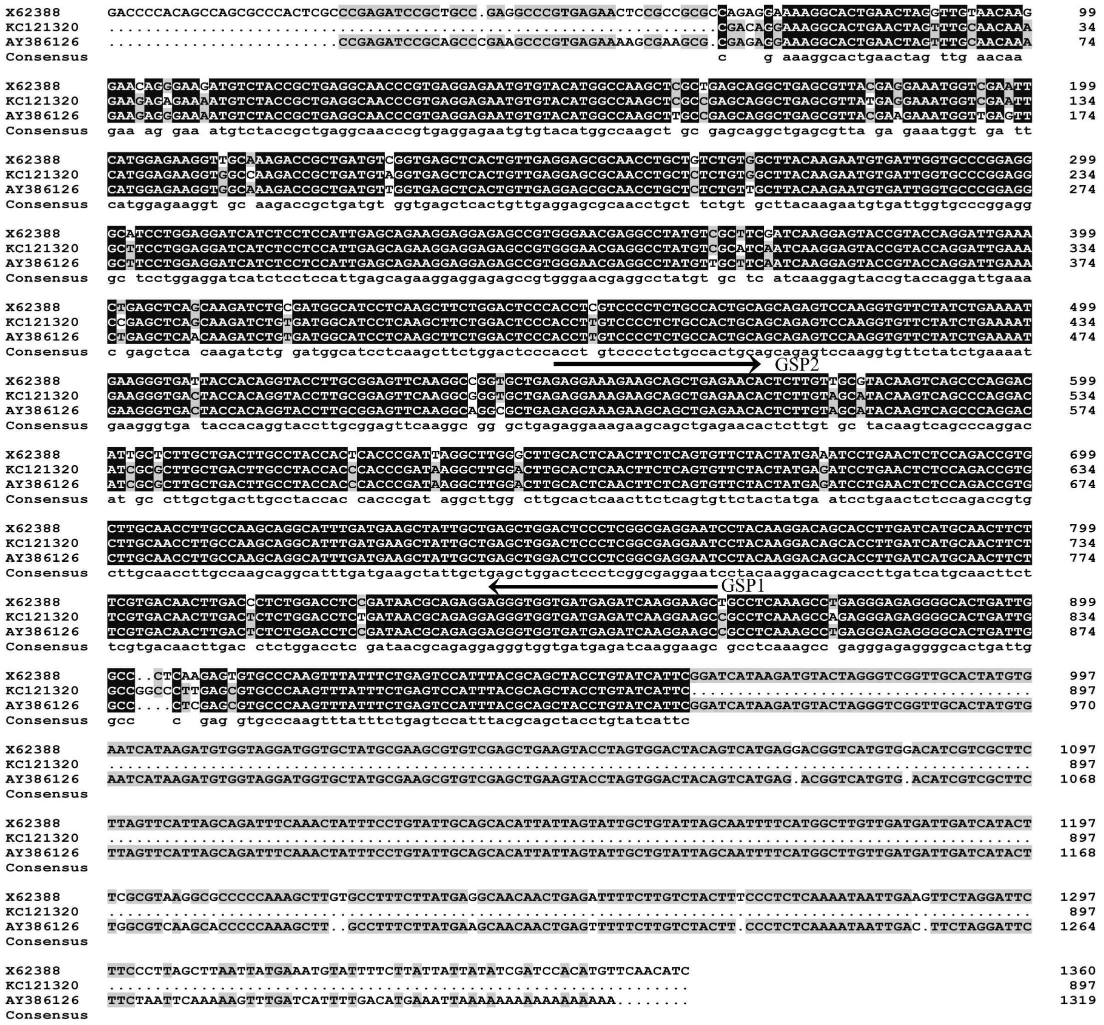

Supplementary Figure S1. Multiple sequence alignment of amino acid sequences of X62388, KC121320 and AY386126 and primer design

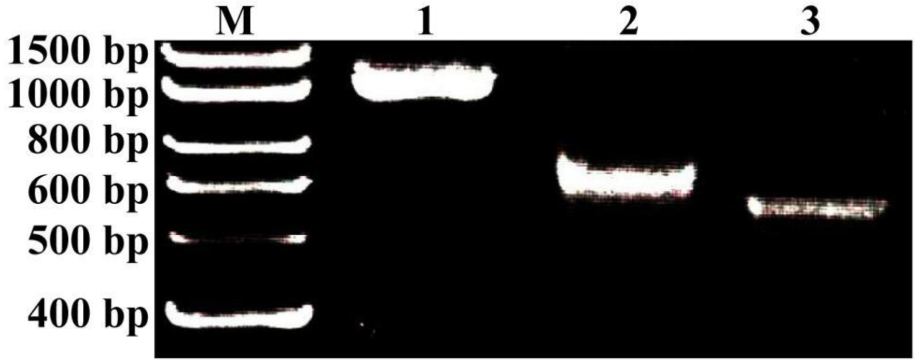

Supplementary Figure S2. PCR amplification of *TaGF14-JM22*. 1, The full length of *TaGF14-JM22*. 2, 3' RACE amplification of *TaGF14-JM22*. 3, 5' RACE amplification of *TaGF14-JM22*.

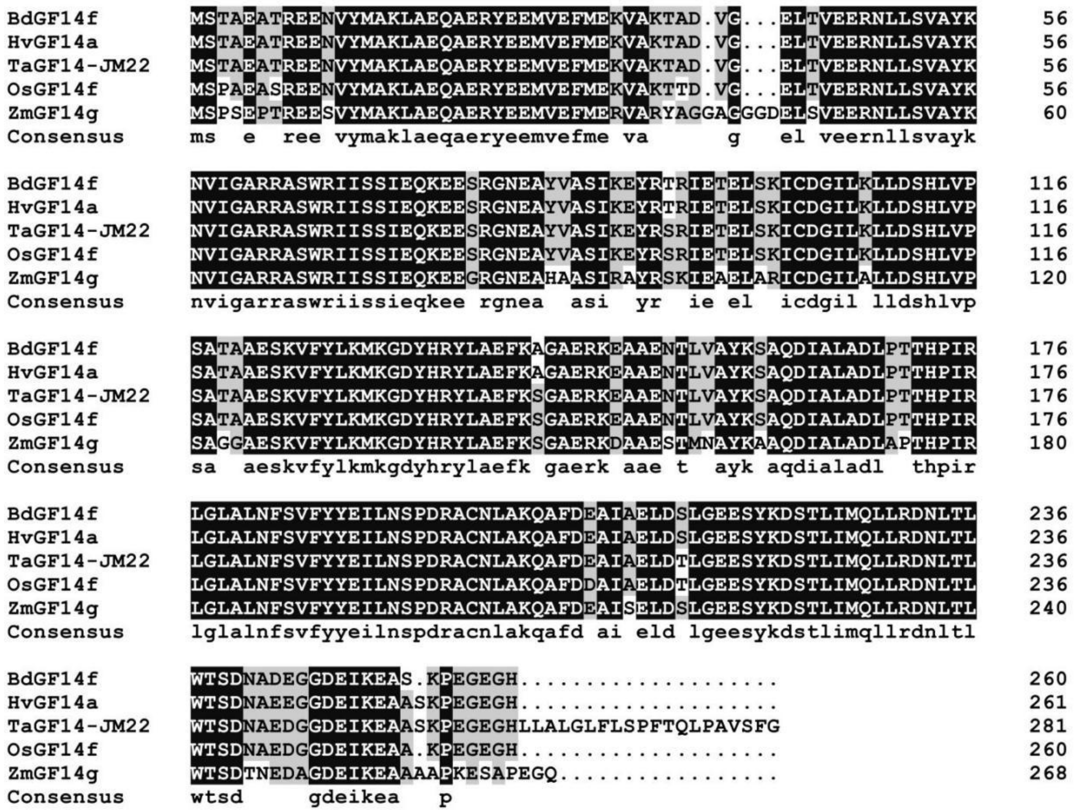

Supplementary Figure S3. Multiple sequence alignment of the amino acid sequences of *TaGF14-JM22* (GenBank JF957590) with four other species, i.e., barley (*HvGF14a*, X62388), rice (*OsGF14f*, AK103065), maize (*ZmGF14g*, *GRMZM2G106424*) and *Brachypodium* (*BdGF14f*, *Bradi1g11290*). Protein sequences were aligned using the CLUSTALW alignment algorithm. Sequences were shaded using the BoxShade program. Identical and conserved residues are shaded in black and grey, respectively. In the picture, *TaGF14-JM22* shows high identity with other *GF14s*.

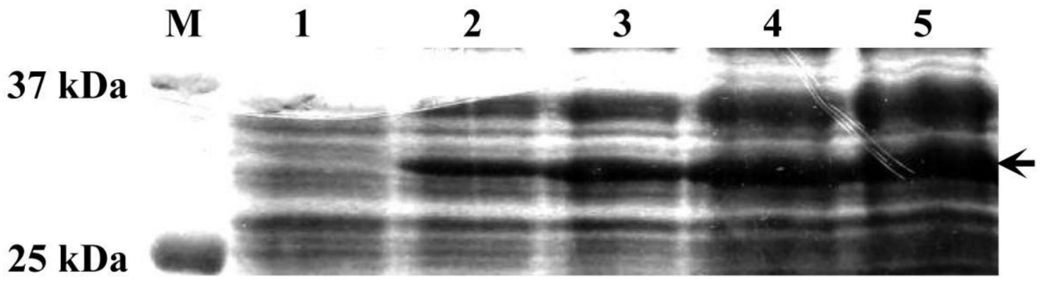

Supplementary Figure S4. Expression and purification of *TaGF14-JM22* in *E coli*. M, Protein markers. 1, Bacterial proteins form BL21 transformed with pMD19-T-*TaGF14-JM22* uninduced with IPTG, lanes 2 to 5, Bacterial proteins from BL21 transformed with pMD19-T-*TaGF14-JM22* induced with 1 mM IPTG at 7 °C for 1, 3, 5 and 7 h, respectively.

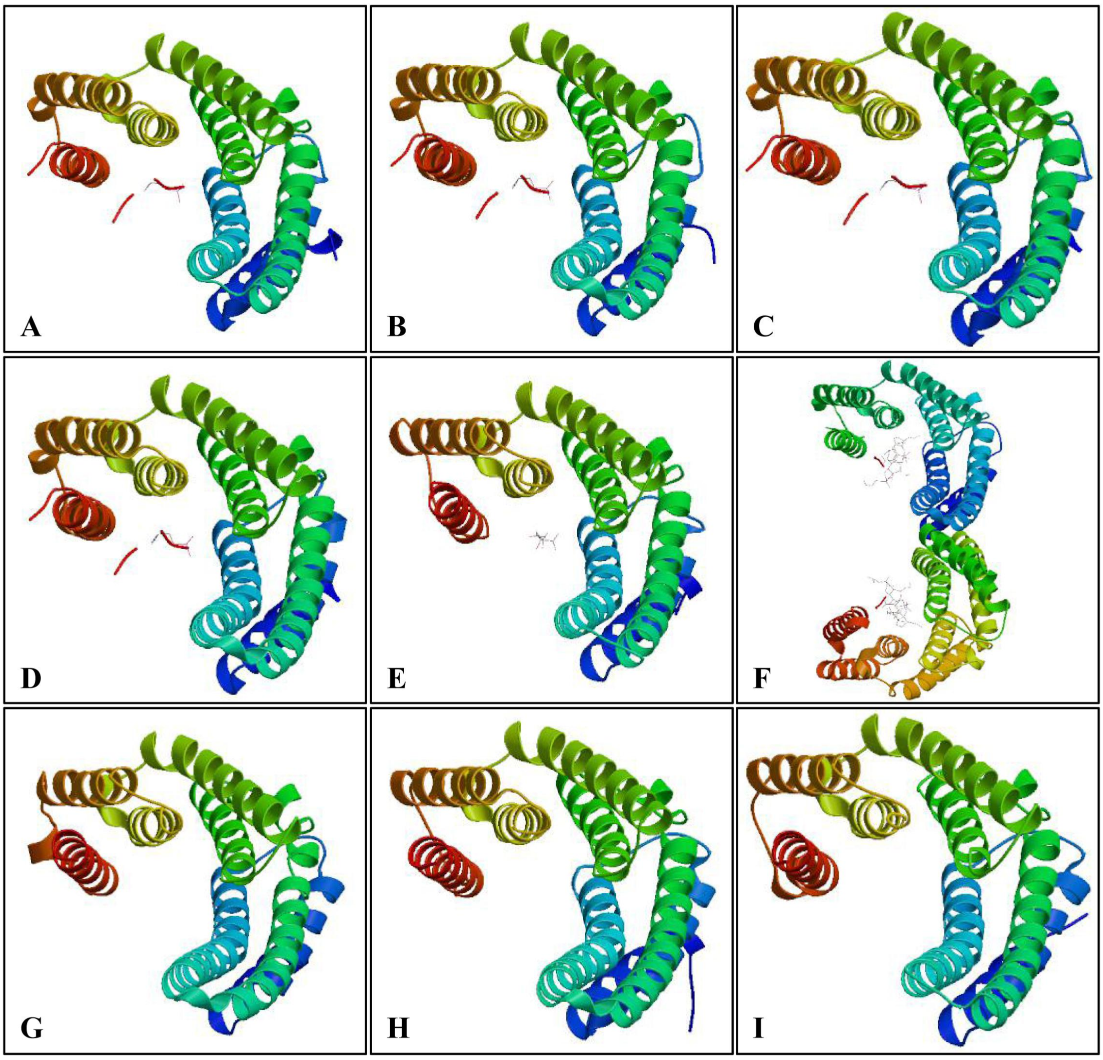

Supplementary Figure S5. The predicted three-dimensional structures of the *TaGF14-JM22* protein. A-I, the QMEAN Z-score evaluations for the models were -1.12, -0.95, -1.50, -1.03, -0.50, -0.97, -1.33, -1.39, and -1.75, respectively.

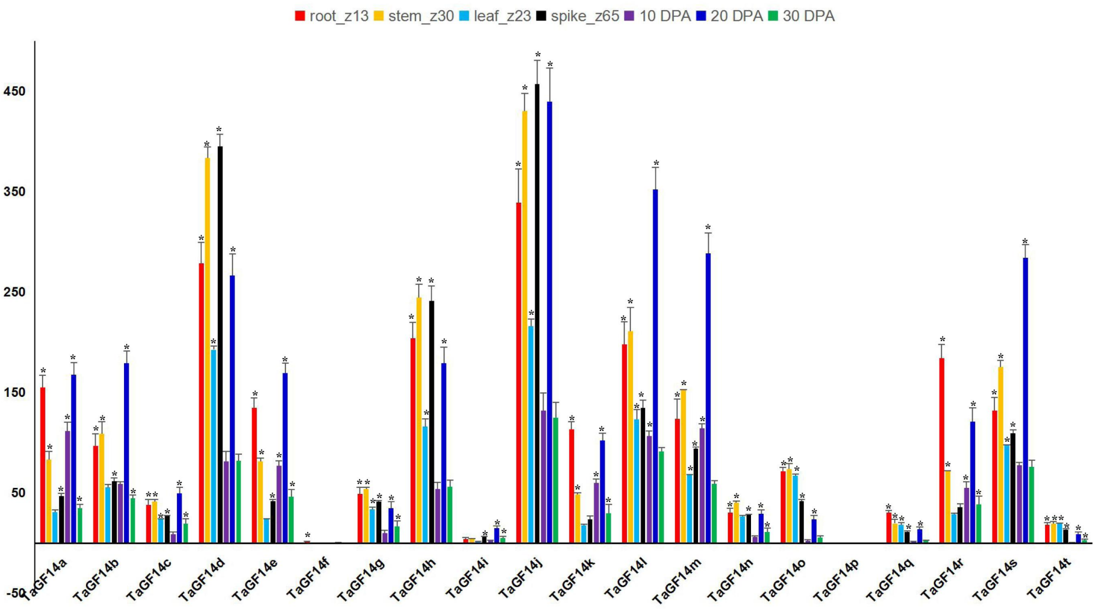

Supplementary Figure S6. Tissue specific expresson of twenty TaGF14s in different tissues. * at the top of each column indicates significant difference at P = 0.05.

Supplementary Tables

Supplementary Table S1. The CDS of 14-3-3 genes and their deduced proteins in 8 representative species

Supplementary Table S2. Predicted prosites of the predicted structure of the 14-3-3 protein from developing wheat endosperms according to the ProtParam tool.

Supplementary Table S3. The primers used in this study.

Reference

Neuhoff, V., Arold, N., Taube, D., Ehrhardt, W. (1988). Improved staining of proteins in polyacrylamide gels including isoelectric focusing gels with clear background at nanogram sensitivity using Coomassie Brilliant Blue G-250 and R-250. *Electrophoresis* 9(6), 255-262.

Tetlow, I.J., Beisel, K.G., Cameron, S., Makhmoudova, A., Liu, F., Bresolin, N.S., Wait, R., Morell, M.K., and Emes, M.J. (2008). Analysis of protein complexes in wheat amyloplasts reveals functional interactions among starch biosynthetic enzymes. *Plant Physiol.* 146 (4), 1878-1891.
